# Alcohol Septal Ablation in Patients with Hypertrophic Obstructive Cardiomyopathy: A Contemporary Perspective

**DOI:** 10.3390/jcm12082810

**Published:** 2023-04-11

**Authors:** Felice Gragnano, Francesco Pelliccia, Natale Guarnaccia, Giampaolo Niccoli, Salvatore De Rosa, Raffaele Piccolo, Elisabetta Moscarella, Enrico Fabris, Rocco Antonio Montone, Arturo Cesaro, Italo Porto, Ciro Indolfi, Gianfranco Sinagra, Pasquale Perrone Filardi, Giuseppe Andò, Paolo Calabrò

**Affiliations:** 1 Department of Translational Medical Sciences, University of Campania “Luigi Vanvitelli”, 83043 Naples, Italy; 2 Division of Clinical Cardiology, Azienda Ospedaliera di Rilievo Nazionale “Sant’Anna e San Sebastiano”, 81100 Caserta, Italy; 3 Department of Cardiovascular Sciences, University Sapienza, 00185 Rome, Italy; 4 Department of Medicine and Surgery, University of Parma, 43121 Parma, Italy; 5 Division of Cardiology, Department of Medical and Surgical Sciences, Magna Graecia University of Catanzaro, 88100 Catanzaro, Italy; 6 Department of Advanced Biomedical Sciences, University of Naples Federico II, 80138 Naples, Italy; 7 Cardiothoracovascular Department, Azienda Sanitaria Universitaria Giuliano Isontina (ASUGI), University of Trieste, 34127 Trieste, Italy; 8 Department of Cardiovascular Medicine, Fondazione Policlinico Universitario A. Gemelli IRCCS, 00168 Rome, Italy; 9 Dipartimento CardioToracoVascolare, Ospedale Policlinico San Martino IRCCS, 16132 Genova, Italy; 10 Mediterranea Cardiocentro, 80122 Naples, Italy; 11 Department of Clinical and Experimental Medicine, University of Messina, AOU Policlinic “G. Martino”, 98122 Messina, Italy

**Keywords:** alcohol septal ablation, myectomy, hypertrophic cardiomyopathy

## Abstract

Alcohol septal ablation is a minimally invasive procedure for the treatment of left ventricular outflow tract (LVOT) obstruction in patients with hypertrophic obstructive cardiomyopathy (HOCM) who remain symptomatic despite optimal medical therapy. The procedure causes a controlled myocardial infarction of the basal portion of the interventricular septum by the injection of absolute alcohol with the aim of reducing LVOT obstruction and improving the patient’s hemodynamics and symptoms. Numerous observations have demonstrated the efficacy and safety of the procedure, making it a valid alternative to surgical myectomy. In particular, the success of alcohol septal ablation depends on appropriate patient selection and the experience of the institution where the procedure is performed. In this review, we summarize the current evidence on alcohol septal ablation and highlight the importance of a multidisciplinary approach involving a team of clinical and interventional cardiologists and cardiac surgeons with high expertise in the management of HOCM patients—the Cardiomyopathy Team.

## 1. Introduction

Alcohol septal ablation is a minimally invasive procedure for treating left ventricular outflow tract obstruction (LVOTO) in patients with hypertrophic obstructive cardiomyopathy (HOCM) who are symptomatic despite optimal medical therapy. The intervention causes a controlled myocardial infarction of the basal portion of the interventricular septum by the injection of absolute alcohol to reduce LVOTO and improve the patient’s hemodynamics and symptoms. Numerous studies have demonstrated the efficacy and safety of the procedure, which today represents a valid alternative to surgical myectomy. The success of alcohol septal ablation depends on patient selection and the experience of the operators and institution where it is performed. In this review, we provide an updated overview of available evidence on alcohol septal ablation, focusing on the importance of a multidisciplinary approach involving a team of clinical and interventional cardiologists and cardiac surgeons with high expertise in managing HOCM patients—the Cardiomyopathy Team.

### Clinical Characteristics of Hypertrophic Cardiomyopathy

Hypertrophic cardiomyopathy (HCM) is a relatively common genetic cardiomyopathy characterized by increased left ventricular wall thickness that is not solely due to hemodynamic overload [1,2]. More than two decades ago, the CARDIA study estimated the prevalence of the disease in young adults to be around 1:500 [3]. More recently, advances in cardiac imaging and genetic testing have allowed the prevalence of HCM to be redefined to approximately 1:200 in contemporary cohorts [4]. Genetic transmission is mostly autosomal dominant and is caused by mutations in cardiac sarcomeric protein genes, with beta-myosin heavy chain (MYH) and myosin-binding protein C (MYBPC3) involved in 70% of cases. Other less common genes are also involved in the case of metabolic (i.e., Anderson–Fabry disease), mitochondrial, and infiltrative disorders (i.e., hereditary cardiac transthyretin-related amyloidosis) [2,5,6,7].

In daily practice, HCM is usually suspected when there is a family history of cardiomyopathy or sudden cardiac death (SCD). Commonly reported symptoms include angina, dyspnea, syncope, and palpitations, which may be due to the presence of left ventricular outflow tract obstruction (LVOTO), atrial fibrillation, ventricular arrhythmias, and heart failure [1,2,5,8]. The diagnosis is confirmed by imaging techniques such as echocardiography or cardiac magnetic resonance (CMR) that show left ventricular wall thickness ≥ 15 mm (or ≥13 in the case of a confirmed gene mutation or affected first-degree relatives) in any myocardial segment. Importantly, these techniques allow the assessment of the left ventricular hypertrophy morphology (i.e., asymmetric, mid-ventricular), LVOTO, concomitant valve disease, and degree of myocardial fibrosis (i.e., CMR with late gadolinium enhancement), which are key features in predicting the risk of SCD [1,9,10,11]. Noteworthy, although some patients with HCM remain long asymptomatic, a non-negligible proportion of cases may initially manifest with life-threatening arrhythmias and SCD, especially in athletes and the young. Therefore, a systematic assessment of multiple parameters (i.e., using the HCM Risk-SCD score) is crucial to predicting and preventing fatal arrhythmias [9].

## 2. Diagnostic and Therapeutic Work-Up in Patients with HCM and LVOTO

### 2.1. Pathophysiology

The presence of LVOTO is a hallmark of HCM manifestation and is a key element in the diagnosis and management of these patients. LVOTO occurs at rest in about 35% of patients with HCM; in approximately 30% of cases, the dynamic obstruction can be provoked during the Valsalva maneuver or exercise [12]. In patients with HOCM, the obstruction is primarily caused by the systolic anterior motion (SAM) of the mitral valve leaflets. This phenomenon is generally attributed to the Venturi effect and the reduction of the mitro-aortic angle and is considered severe when it occupies more than 30% of the systolic phase [1,2,13,14,15]. Another critical factor in the development of LVOTO is the presence of mitral valve and papillary muscle abnormalities (i.e., mitral leaflets elongation, abnormal chordal attachment, and anterior papillary muscle rotation/displacement) [1,16]. As the LVOTO is often dynamic, it may vary according to loading conditions, left ventricular contractility, and respiratory cycle. Therefore, these aspects should be considered in therapeutic decision-making [13,17,18].

### 2.2. Diagnosis

The diagnosis of HOCM is usually made by echocardiography and is defined in the presence of a peak LVOT gradient ≥ 30 mmHg measured by continuous Doppler (at rest or after the Valsalva maneuver). The LVOT gradient becomes hemodynamically relevant when its peak reaches ≥ 50 mmHg, identifying individuals who are candidates for invasive septal reduction therapies. Notably, when patients with a peak gradient ≤ 50 mmHg at rest present with suggestive symptoms, a physical stress echocardiography is indicated to identify a latent obstruction [1,2]. Pharmacological stress tests with dobutamine or nitrates are usually poorly tolerated and may not reproduce the true mechanism of obstruction; therefore, they are generally discouraged and reserved for selected cases [19,20]. The prognostic value of LVOTO remains controversial, considering that fatal events occur with similar incidence in patients with and without obstruction. Accordingly, whether the presence and the grade of obstruction should only be considered as a clinical/hemodynamic marker or as a prognostic factor remains controversial [21].

### 2.3. Therapeutic Work-Up

Medical therapy is the first-line treatment for patients with HOCM, with the goal of reducing obstruction and relieving symptoms. Non-vasodilating β-blockers (i.e., propranolol, nadolol, bisoprolol, and metoprolol) are the first choice. In case of intolerance, current guidelines recommend non-dihydropyridine calcium channel blockers (i.e., verapamil and diltiazem). In patients who are intolerant or poor-responders to these drugs, disopyramide should be considered because of its negative inotropic effect. Allosteric myosin inhibitors (i.e., mavacamten and aficamten) have recently been proposed as new therapeutic strategies in HOCM patients to prevent/delay the need for invasive treatment [1,2,22,23,24,25]. Specific to mavacamten, the first U.S. Food and Drug Administration (FDA)-approved medication targeting HCM, the EXPLORER-HCM (Clinical Study to Evaluate Mavacamten [MYK-461] in Adults With Symptomatic Obstructive Hypertrophic Cardiomyopathy) trial [26] and the VALOR-HCM (A Study to Evaluate Mavacamten in Adults With Symptomatic Obstructive HCM Who Are Eligible for Septal Reduction Therapy) trial [27] have shown that a significant proportion of HCM patients experience an improvement in clinical endpoints and quality-of-life measures. In a further subgroup of patients, mavacamten significantly reduced the fraction of patients meeting guideline criteria for septal reduction therapy after 16 weeks.

Septal reduction therapy has clearly shown to be effective in reducing LVOTO and should be considered in HOCM patients with LVOT gradient > 50 mmHg, moderate to severe symptoms and/or exertional syncope despite maximally tolerated medical therapy [1]. The septal reduction can be completed by either surgical myomectomy or percutaneous catheter-based intervention. The first percutaneous septal reduction treatment was performed with alcohol septal ablation. Subsequently, nonalcohol agents, including microspheres, have been proposed as an alternative to alcohol. More recently, radiofrequency ablation has been used for non-surgical septal reduction as it is both minimally invasive and independent of coronary anatomy. This novel technique uses echocardiography to guide the transapical placement of an intraseptal radiofrequency electrode that delivers energy to the core of the hypertrophic segment. The authors reported an impressive septal reduction of 11 mm with a resolution of LVOTO and improvements in functional class [28].

## 3. Alcohol Septal Ablation

### 3.1. Historical, Clinical, and Procedural Considerations

The concept of alcohol septal ablation in patients with HOCM was first introduced in the 1980s by Gunnar Berghöfer (March 1989, personal communication), based on several studies on the effects of temporary balloon occlusion of coronary arteries on myocardial wall motion and alcohol trans-coronary ablation of ventricular tachycardia [29,30]. In 1995, a German cardiologist, Ulrich Sigwart, first described three cases of HOCM patients who were persistently symptomatic despite optimal medical therapy and were treated with alcohol septal ablation. All patients had a complete resolution of the LVOTO and regression of symptoms from the day after the procedure, showing the potential of this strategy [31].

Current European and American guidelines recommend the use of alcohol septal ablation in patients who remain symptomatic on maximally tolerated medical therapy (i.e., New York Heart Association [NYHA] class III-IV), with evidence of an LVOT gradient > 50 mmHg and established contraindications to surgery [1,2]. The procedure consists of the injection of a small amount of absolute alcohol into the septal arteries to induce iatrogenic myocardial infarction selectively localized in the basal part of the interventricular septum. Despite the lack of large-scale randomized trials, numerous observational studies in recent decades have provided sufficient evidence to support the use of this strategy as a viable alternative to surgical myectomy, with the advantage of a shorter hospital stay and rapid discharge [1].

The first steps in evaluating symptomatic HOCM patients who are candidates for alcohol septal ablation are careful medical history collection and clinical examination to exclude other conditions that may act as possible confounders (i.e., coronary artery disease, respiratory disease, and anemia). Among demographic factors, age should be considered in the decision-making: although there is no specific age cut-off in current guidelines, alcohol septal ablation is generally not the preferred strategy in children and young adults, given the higher rate of LVOTO recurrence and a lack of long-term data on clinical outcomes [1,32,33,34]. Other important elements are represented by comorbidities and patient frailty, which may contraindicate surgery in favor of a less invasive approach. The presence of other surgical indications (coronary artery disease and valvular heart disease) also favors surgical myectomy, whereas the history of cardiac surgery favors alcohol septal ablation. The baseline ECG should also be considered as both surgical and non-surgical interventions can be complicated by rhythm disturbances, including complete atrioventricular block. Because the infarct is located near the right bundle branch, the development of a right bundle branch block is common after alcohol septal ablation. Therefore, this procedure is not recommended in the case of pre-existing left bundle branch block (unless a pacemaker has been previously implanted). On the other hand, as surgical myectomy can be associated with the development of a left bundle branch block, alcohol septal ablation is favored in patients with pre-existing right bundle branch block [35,36,37]. Finally, comprehensive morphological and angiographic assessment by cardiac ultrasound, CMR, CT-angiography, and/or invasive coronary angiography is essential to assess whether the patient can successfully undergo the procedure.

### 3.2. Pre-Procedural Anatomic Evaluation

Left ventricular and mitral valve anatomy should be carefully assessed before the intervention. Imaging assessment includes LVOT geometry, extent and distribution of myocardial hypertrophy and fibrosis, septal morphology, and mitral valvular and subvalvular anatomy, all crucial in predicting procedural success. In this context, CMR provides high-resolution images and should be used routinely in the pre-procedural anatomic assessment of patients undergoing an invasive treatment for HOCM [38]. Abnormalities of the mitral valve apparatus and papillary muscles can significantly reduce the procedural success of alcohol septal ablation and should be excluded by comprehensive imaging assessment. Conversely, the procedure can be performed in case of posterior mitral regurgitation secondary to systolic anterior motion of the anterior mitral leaflet. The presence of a focal *septal bulge*, a wide angle of the papillary muscles, and chords to the ventricular septum also favor alcohol septal ablation, whereas midventricular hypertrophy leans toward surgical myectomy. A septal thickness of ≥17 mm is a widely accepted cut-off to safely perform alcohol septal ablation and minimize the risk of an iatrogenic ventricular septal defect [1,16]. However, the procedure may be suboptimal in the case of severe hypertrophy (>25 mm), possibly because of the need for high-dose alcohol infusion and the subsequent increased risk of complications. Furthermore, high LVOT gradient (>100 mmHg), large left atrial diameter (>40 mm), and low center experience (less than 50 patients) are additional predictors of poor post-procedural outcomes [35,39,40].

The key to successful alcohol septal ablation is suitable coronary anatomy and the correct selection of the septal branches. The diameter of the left anterior descending artery (LAD) has been reported to be an independent predictor of successful ablation, with a smaller vessel being associated with a higher likelihood of success [40]. The first septal branch often perfuses the basal septum (which is commonly involved in LVOTO) and is often the target vessel for ablation. This vessel usually originates from the LAD and runs close to the His bundle and right bundle branch, although, in 15% of cases, it may originate from other arteries such as the diagonal, the ramus intermedius, the left main, or even from the right coronary artery [41,42,43,44]. The complex anatomical variability mandates a careful and systematic assessment of the coronary tree for a safe procedure. The most appropriate coronary anatomy consists of a single septal perforator of adequate size supplying the target area. In the case of multiple septal branches supplying the hypertrophic basal septum, all should be ablated during the index or in staged procedures, if necessary. Yet, in 10–15% of patients, a culprit septal branch for ablation cannot be identified, forcing the operator to abandon the procedure [41,45]. Importantly, septal branches may supply other myocardial segments, such as the free walls of the left and right ventricles or papillary muscles. This condition is an absolute contraindication to alcohol injection because of the risk of potentially life-threatening complications such as extensive myocardial infarction. Intracoronary contrast echocardiography performed during the procedure is recommended in all patients undergoing ASA to ensure the size and the localization of iatrogenic myocardial infarct, prevent adverse events, and assess procedural success [36].

### 3.3. Description of the Procedure

**Figure 1:**
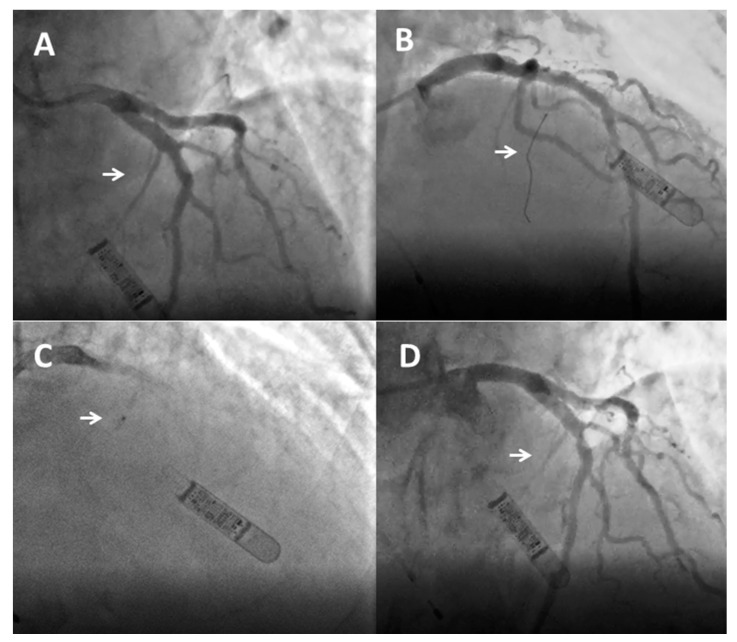
Angiographic sequence of alcohol septal ablation. (**A**) Baseline angiogram of the left coronary artery with estimated target septal branch (arrow). (**B**) Wiring of the target septal branch (arrow). (**C**) Injection of angiographic contrast media through the lumen of the over-the-wire balloon (arrow). (**D**) Occlusion of the distal portion of the septal branch (arrow) after balloon retraction 10 min after the last alcohol injection without damage to the left anterior descending artery.

Alcohol septal ablation consists of selective infusion of 95–96% absolute alcohol into the septal perforator branch supplying the left ventricular side of the basal or mid cavitary septum [43,46]. The rationale is to create an alcohol-induced occlusion of the vessel, with a controlled infarct in the basal septum that progressively changes from viable hypertrophic myocardium to a thin akinetic scar, thus reducing LVOTO. Radial and femoral access are both feasible, and the choice mainly depends on operator preference. In fact, the two approaches show similar short- and long-term success rates, although the radial approach has been associated with fewer vascular complications [47]. The main steps of the procedure are shown in Figure 1. After placement of a 6–7 Fr arterial sheath and temporary pacemaker via the femoral or internal jugular vein, analgesics (i.e., morphine) can be administered to control pain caused by alcohol injection and iatrogenic infarction. Coronary angiography is then performed to select the septal branch for ethanol infusion and to assess vessel anatomy, origin, angulation, and size. The course of septal vessels can be appropriately visualized using the right anterior oblique or postero-anterior cranial projections, while the left anterior oblique view allows differentiation of whether the septal branches run along the right or left side of the septum (the selection of left-sided branches reduces the risk of an atrioventricular block) [43,46].

**Figure 2:**
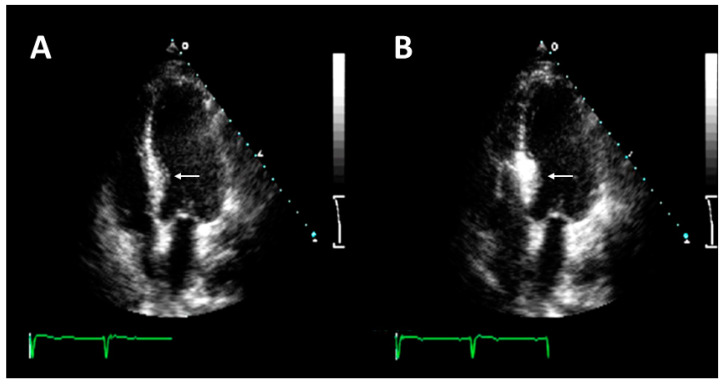
Echocardiographic contrast used for the guidance of alcohol septal. (**A**) Four-chamber echocardiogram shows increased LV wall thickness at basal septum level with no apical aneurysm (arrow). (**B**) Intracoronary echocardiographic contrast injection is localized to the basal septum at the site of greatest hypertrophy (arrow) without extending beyond the point of mitral septal contact, consistent with an appropriate target vessel for alcohol septal ablation.

After engaging the left main with a guide catheter providing extra support, a short over-the-wire (OTW) (1.5–2.5 mm in diameter, 6–10 mm long, with a balloon-to-artery ratio of approximately 1.3:1) is passed over a standard 180 cm 0.014″ extra support wire and positioned in the target vessel. OTW balloons are recommended as they allow selective septal branch angiography during balloon inflation (1–2 mL of contrast slowly injected into the proximally occluded vessel) to verify the correct positioning, complete septal occlusion, and absence of contrast reflux into the LAD. During balloon inflation, continuous invasive monitoring may reveal a reduction in LVOT pressure gradient, indicating a good target vessel for ablation [44]. Due to the high degree of collateralization between the left and right coronary arteries, it is imperative to exclude the filling of other coronary arteries by septal collaterals prior to alcohol injection [48]. The target vessel must then be tested using myocardial contrast echocardiography (Figure 2): 1–2 mL of contrast medium should be injected through the OTW balloon to visualize the target area at the basal septum, adjacent to the point of mitral-septal contact, and to rule out contrast enhancement in other regions (i.e., inferior wall, papillary muscles, and right ventricle), which is an absolute contraindication to ethanol infusion and requires interruption of the procedure [49]. In more challenging cases, intracardiac or 3D contrast echocardiography may be helpful for intraprocedural guidance [50]. The operator can then inject ethanol over 1 to 5 min [1]. The amount of alcohol is about 0.7–1 mL per 10 mm of measured septal thickness [51]. During ethanol infusion, the inflated balloon must be firmly placed to completely occlude the vessel and avoid extensive myocardial damage due to reverse flow in the LAD or other coronary vessels [50]. The aggressive injection is discouraged as ethanol may pass through collaterals and cause inferior wall injury. Finally, analgesic infusion (i.e., morphine) is recommended immediately before alcohol infusion to control the pain caused by the alcohol injection and the provoked ischemia. The final coronary angiography should be performed about 20 min after alcohol infusion to exclude possible complications and conclude the procedure.

### 3.4. How to Assess Procedural Success?

The goal of alcohol septal ablation is to achieve a significant and sustained improvement in symptoms and to reduce LVOT gradient by >50%. Additional beneficial effects include a reduction in mitral regurgitation and left ventricular end-diastolic pressure, which results in a lower incidence of atrial fibrillation and an improvement in pulmonary hypertension [36,52]. Favorable cardiac remodeling is also an important effect of the procedure, particularly in young patients, and may be clinically evident after 12 months [33,53]. Among possible predictors of procedural failure, a total creatine kinase (CK) peak less than 1300 U/L and an immediate residual LVOT gradient greater than 25 mm Hg have been reported in previous studies, although their association with long-term clinical outcomes remains uncertain and requires further investigations [37,54,55,56,57]. To date, there is no standardized follow-up pathway after alcohol septal ablation; therefore, the follow-up schedule usually depends on the center’s experience. In the case of an uncomplicated post-procedural course, hospital discharge is usually 3–5 days after ablation [58]. After discharge, the patient should preferably be evaluated at one month, three months, and one year to assess changes in LVOT gradient. Notably, while some patients experience “monophasic” success (≥50% gradient reduction at three days and three months), in some cases, “triphasic” success can be observed (<50% gradient reduction at three days but ≥50% gradient reduction at three months or later) [59]. Therefore, serial evaluations should be performed to monitor the short- and long-term evolution of hemodynamic changes after the procedure.

Numerous studies have shown that approximately 90% of patients undergoing a successful procedure experience an improvement of functional status (i.e., post-procedural NYHA class I-II), and in 80% of cases, there is a significant reduction in LVOTO [53]. CMR can be used during the follow-up to quantify the size and location of the iatrogenic infarct. The use of CMR is also helpful in the case of procedural failure to evaluate the reasons for unsuccess (i.e., the iatrogenic infarct is too small or outside the target area) [60]. In the event of failure, surgical myectomy may be performed as a rescue strategy. Of note, several studies have reported on patients with a previous alcohol septal ablation undergoing surgical myectomy, showing that these patients have a higher risk of complete atrioventricular block and progression to heart failure because of the more extensive conduction system and myocardial injury [60,61,62].

### 3.5. Procedural Safety and Possible Complications

A complete atrioventricular block is the most common complication after alcohol septal ablation (transient in about 30% of patients and permanent in about 10%) and is due to the alcoholic injury to the conduction system [36,53]. This adverse event may occur during the procedure or in the first few days after and is more frequent in older patients or those with preexistent conduction disorders [33]. In patients with pre-existing left bundle branch block, a temporary pacemaker could be placed after the procedure, while a permanent pacemaker is indicated if an advanced block persists for more than 24–72 h [43,46]. If a concomitant indication for an implantable cardiac defibrillator exists, device implantation should precede the procedure to simplify the management of post-procedural arrhythmias. Infarction of the left and right ventricle-free walls or papillary muscles is a possible but rare adverse event related to the presence of collateral branches supplying distant myocardial areas and to the reflow of alcohol in the LAD artery. Although there have been concerns in the past about the arrhythmic risk associated with the septal scar, recent studies have demonstrated the long-term safety of the procedure, with a survival rate similar to surgical myectomy [52,63]. The rate of early mortality (up to 30 days) is relatively low and approximates 1.5%. Causes of death include LAD dissection, ventricular fibrillation, cardiac tamponade, cardiogenic shock, and pulmonary embolism. Late mortality is reported in 0.5% of patients and is often due to SCD, heart failure, pulmonary embolism, or other non-cardiac causes [36].

Alternative techniques have been explored to minimize the risks associated with the potential spillover of alcohol outside the target area, including various embolization techniques [64,65,66,67]. The most promising is the use of (n-butyl cyanoacrylate), a clear and colorless monomer that polymerizes rapidly on contact with blood [68]. Initial clinical experience with cyanoacrylate for septal ablation in HOCM patients showed an excellent safety profile, paving the way for long-term efficacy studies [69]. In addition, several catheter-based procedures, including the PIMSRA (percutaneous intramyocardial septal radiofrequency ablation) and the SESAME (septal scoring along the midline endocardium), have recently been tested in patients ineligible for surgery and alcohol septal ablation with encouraging results [70,71]. However, these procedures are currently performed in a limited number of centers in the US, Europe and Asia, and further prospective data are needed to safely introduce such novel strategies into practice.

### 3.6. Comparison between Alcohol Septal Ablation and other Surgical Reduction Therapies

Despite extensive research, the choice of the best option for septal reduction strategy in the individual patient remains challenging and poses numerous clinical dilemmas. To date, there is no current or completed randomized trial comparing surgical myectomy vs. alcohol septal ablation, and all available information is derived from retrospective investigations. Overall, the benefits of alcohol septal ablation are comparable to those seen with surgical myectomy in terms of functional class, exercise capacity, and LVOTO regression. Morbidity and mortality resemble those of surgical intervention. The major complication of alcohol septal ablation compared with surgery is a complete atrioventricular block requiring pacemaker implantation and the need for a re-do procedure. At variance with surgery, there are poor data on the comparison between alcohol septal ablation and other catheter-based interventions or the novel pharmacologic therapy with allosteric myosin inhibitors.

The lack of randomized evidence affects existing recommendations which are primarily based on observational findings and expert consensus. European and American guidelines do not provide class I recommendations for any of the invasive options [1,2] and the choice in the individual patient is largely determined by clinical judgement, local expertise, and patient preference. Specifically, the 2014 European guidelines are not in favor of any procedure but highlight that alcohol septal ablation is controversial in children, adolescents, and young adults for the absence of data on the long-term effects of a myocardial scar in these groups [1]. Conversely, the 2020 American guidelines for the diagnosis and treatment of HOCM state that myectomy should be preferred over alcohol septal ablation [2]. However, they recommend the latter procedure—when feasible and performed in experienced centers—in adult patients with symptomatic HOCM in whom surgery is contraindicated or risk is considered unacceptably high because of serious comorbidities or advanced age.

## 4. Multidisciplinary Evaluation and Management: The HCM Heart Team

The choice of the optimal septal reduction strategy (i.e., surgical vs. non-surgical) is crucial in the management of HOCM and should be the result of a comprehensive and personalized evaluation, as the success of the intervention depends on the patient’s characteristics and center expertise. To date, procedural success and long-term survival are comparable between alcohol septal ablation and surgical myectomy when patients are carefully selected, and the procedures are performed in high-volume centers [72,73]. Importantly, the risks and benefits of both procedures should always be discussed with patients in order to match their expectations and preferences [1].

Given the complexity of clinical and anatomical factors that characterize both surgical and non-surgical strategies, it is crucial that the final decision is made by an experienced team working in centers of excellence for the management of HOCM [14,74]. In analogy with the “Heart Team” approach that is the current standard of care for patients with coronary and valvular heart disease [75,76,77,78], the “Cardiomyopathy Team” should represent the standard approach for the management of patients with HOCM. This team should include at least a clinical cardiologist, an interventional cardiologist, and a cardiac surgeon with documented expertise in the treatment of HOCM patients [14,74]. Specifically, the operators should have high experience with a minimum caseload of 10 alcohol septal ablations or surgical myectomies per year, as recommended by current guidelines [1,2]. This concept is supported by the evidence that highly experienced operators/institutions have a lower incidence of complications, a higher success rate, and a lower rate of re-intervention [79]. To date, further studies are needed to define better the role of the Cardiomyopathy Team in clinical practice and inform international guideline recommendations in this regard.

## 5. Conclusions

Alcohol septal ablation is a minimally invasive procedure for treating patients with HOCM who remain symptomatic despite optimal medical therapy. Available evidence supports the use of this procedure as a valid alternative to surgical myectomy when performed in high-volume centers. The appropriate patient selection remains critical in the decision-making process to maximize procedural success and minimize the risk of complications. Today, a precise and multidisciplinary assessment by the Cardiomyopathy Team appears to be crucial to improving the management and prognosis of patients with HOCM in daily practice.

## Data Availability

Not applicable.
